# FAK loss reduces BRAFV600E-induced ERK phosphorylation to promote intestinal stemness and cecal tumor formation

**DOI:** 10.21203/rs.3.rs-2531119/v1

**Published:** 2023-02-01

**Authors:** Chenxi Gao, Huaibin Ge, Shih-Fan Kuan, Chunhui Cai, Xinghua Lu, Farzad Esni, Robert Schoen, Jing Wang, Edward Chu, Jing Hu

## Abstract

BRAFV600E mutation is a driver mutation in the serrated pathway to colorectal cancers. BRAFV600E drives tumorigenesis through constitutive downstream extracellular signal-regulated kinase (ERK) activation, but high-intensity ERK activation can also trigger tumor suppression. Whether and how oncogenic ERK signaling can be intrinsically adjusted to a “just-right” level optimal for tumorigenesis remains undetermined. In this study, we found that FAK (Focal adhesion kinase) expression was reduced in BRAFV600E-mutant adenomas/polyps in mice and patients. In Vill-Cre;BRAFV600E/+;Fakfl/fl mice, Fak deletion maximized BRAFV600E’s oncogenic activity and increased cecal tumor incidence to 100%. Mechanistically, our results showed that Fak loss, without jeopardizing BRAFV600E-induced ERK pathway transcriptional output, reduced EGFR (epidermal growth factor receptor)-dependent ERK phosphorylation. Reduction in ERK phosphorylation resulted in increased mRNA expression and stability of Lgr4, promoting intestinal stemness and cecal tumor formation. Together, our findings show that a “just-right” ERK signaling optimal for BRAFV600E-induced cecal tumor formation can be achieved via Fak loss-mediated downregulation of ERK phosphorylation.

## Full Text Availability

The license terms selected by the author(s) for this preprint version do not permit archiving in PMC. The full text is available from the preprint server.

